# Monthly Summary

**Published:** 1881-03

**Authors:** 


### MONTHLY SUMMARY.

---

*A Curious Fracture of Skull and Face.*—Dr. Palmer gave a verbal description of the Burr Robbins case as follows: Mr. Robbins's injuries were received Jan. 17, 1880, by coming into collision with a bridge while standing upon a small steamboat which was moving very rapidly. His head was caught between the timbers of the bridge and the flange of the boiler, which was an upright one. The bones of the skull were crushed in; the frontal bone was separated along the course of the sutures, longitudinally and transversely, and raised out in front as far as the tissues would permit; without being torn they were put upon the stretch so that the bone could be raised and lowered like the lid of a box over a space of about two inches. The temporal bone was depressed—crushed in, about an inch and a half back of the eye, and the bone forming the upper part of the orbit of both eyes crushed into fragments, as were also the nasal bone on one side. The bones on the opposite side were adjusted as accurately as possible. There was a fracture of the malar bone extending down toward the throat, and the upper jaw was broken loose, and knocked over toward the left side about one-quarter of an inch, so that the whole jaw might be moved to and fro laterally to that extent. There was also a fracture at the back of the head and a scalp wound of about three and one-half inches in extent. The amount of bone removed was about three and one-half square inches in area. The brain was lifted out of its resting place about half an inch, and one of the internal temporal arteries was torn across. Of course, it seemed as though such injuries must prove fatal almost instantly. Although the patient was a man of remarkable vitality and power, he was at the point of dying about half an hour after the accident. The marked peculiarity of the case was the retention of consciousness by the sufferer. While his wounds were being dressed he gave directions about his business, made his will, and adjusted all his affairs as if nothing were the matter. The only loss of consciousness occurred while a sponge wet with cold water was applied to the surface of the brain. When the pressure was made he would cease talking, and the instant it was removed he would resume at the point where he left off. He has suffered more from the injury to the upper jaw than from the damage to the brain. At present he can use either eye separately, but there is a loss of co-ordinating power which prevents the use of both together. He is in very good physical condition and fair mental power; says that he can attend to his business and give general directions, but tires quickly.

Dr. Vivian also gave details of an interesting case in which the skull of a child had been crushed in by the kick of a horse, and in which there was no loss of consciousness at any time except when under the influence of chloroform for the removal of the bone.—*Med. Record.*

---

*Tharapeutical Effects of Chlorate of Potash.*—Two monographs on this subject from Dr. Harkin's pen have already appeared. The first, entitled, "The use of Chlorate of Potash in the Treatment of Consumption and Scrofula," will be found in the *Dublin Quarterly Journal of Medical Science* for November, 1871; the second, "On Chlorate of Potash in the Hæmorrhage Diathesis," was read before the British Medical Association, at its meeting in Cork, in August, 1877. Dr. Harkin, (*Dub. Med. Jour.*, May 1880,) as the result of observations extending over many years, considers that chlorate of potash exercises a most potent influence on all maladies dependent on defective nutrition and molecular metamorphosis; that it possesses the power of developing vital force in weakened constitutions, of retarding the degeneration of the tissues, and of controling the too rapid advance of senility due to climatic condition. After its continued employment, the patient experiences an increase of appetite, and of nervo-muscular force; all the bodily functions are performed with greater ease, the color improves, and the flesh-producing power, as shown by increase of weight, is augmented. For internal administration, it is ordered in solution—an ounce of salt to twenty of water, and of this two tablespoonfuls should be given three times a day, either before or after meals. It has been prescribed for thousands of patients, with wonderful success, and rarely, if ever, disagrees. In one instance, a young man took by mistake an ounce of the salt at a dose, without experiencing any unpleasant symptoms. In the form of a lotion—five grains to the ounce—it is a valuable application to burns and scalds, the healing process proceeding more rapidly than under other modes of treatment. In caries of the vertebræ, and in strumous abscesses and sinuses, its beneficial effects are very remarkable. As an application to ulcers—simple, irritable, indolent and rodent—it would be difficult to overestimate its value. The hard and elevated edges of old ulcers give way to flattened and healthy ones, and the excavated surface of the sore is altered by the oxygenating power of the lotion, and replaced by healthy granulations.—*The London Medical Record.*

---

*Adulterations in a New Sugar.*—The manufacture of a new kind of sugar by a secret process has excited much attention again to the question of the adulterations of this article. The City Board of Health is now engaged in examining various kinds and grades of sugars for the purpose of obtaining exact knowledge on the subject, and it will doubtless furnish facts that will be decisive as to how much harmful adulteration there really is. Meanwhile, there is a good deal of talk and protest concerning the deceitfulness of the so-called "new process sugar." This sugar is made by mixing very carefully refined grape-sugar with various grades of ordinary cane-sugar. As the grape-sugar is perfectly white, it of course gives a finer appearance to the dark sugars with which it is mixed, and, as it costs only four or five cents a pound, considerable profit can be made. The grape-sugar is made out of corn, sulphuric acid being used in the process. As prepared by the "new process," it is white, dry, very finely grained, and not so sweet as cane-sugar. The only objection that can be made against it is that sulphuric acid and gypsum are liable to become mixed with it in its manufacture. Such adulteration, however, if it exists, is in too small amount to be of any harm. The same may be said of the alleged adulterations of other sugars. It is very doubtful if the Board of Health finds anything to alarm housekeepers as the result of their investigations.—*Med. Record.*

